# Genotype-Specific Root System Remodeling of *Paspalum* spp. Induced by Plant Growth-Promoting Bacteria Enhances the Potential for Sustainable Tropical Pastures

**DOI:** 10.3390/plants15162465

**Published:** 2026-08-14

**Authors:** Beatriz Cortellini Ferranti, Gabriel Silva Guimarães, Artur Berbel Lirio Rondina, Marcelo Mattos Cavallari, Alessandra Pereira Favero, Marco Antonio Nogueira, Mariangela Hungria

**Affiliations:** 1 Department of Biochemistry and Biotechnology, Universidade Estadual de Londrina, PR-445, km 380, P.O. Box 10.011, Londrina 86057-970, Paraná, Brazil; cf.beatriz@hotmail.com (B.C.F.); gabrielsguimaraes@hotmail.com (G.S.G.); 2 Soil Biotechnology Laboratory, Embrapa Soja, P.O. Box 4006, Londrina 86085-981, Paraná, Brazil; marco.nogueira@embrapa.br; 3 CNPq, Conselho Nacional de Desenvolvimento Científico e Tecnológico, SHIS QI 1 Conjunto B, Lago Sul, Brasília 71605-001, Federal District, Brazil; arturrondina@hotmail.com; 4 Embrapa Pecuária Sudeste, Rod. Washington Luiz, Km 234, São Carlos 13560-970, São Paulo, Brazil; marcelo.cavallari@embrapa.br (M.M.C.); alessandra.favero@embrapa.br (A.P.F.)

**Keywords:** *Azospirillum brasilense*, *Pseudomonas fluorescens*, PGPB, root morphological traits, tropical pastures

## Abstract

Brazilian cattle production relies predominantly on pasture systems, many of which are degraded by nutrient depletion, particularly nitrogen deficiency. Plant growth-promoting bacteria (PGPB) offer a sustainable strategy to improve pasture productivity, but their effects are often host-specific and remain poorly explored in *Paspalum*. This study evaluated the effect of inoculation with *Azospirillum brasilense* strains CNPSo 2083 (=Ab-V5) and CNPSo 2084 (=Ab-V6) and *Pseudomonas fluorescens* strain CNPSo 2719 (=CCTB 03), applied by seed inoculation or leaf spray, on the root morphology and shoot growth of four *Paspalum* accessions under controlled axenic greenhouse conditions, evaluated in an early growth stage at 35 days after emergence. Genotype-specific responses were the central finding of this study: *Paspalum malacophyllum* BGP-293 showed negligible responses to inoculation, whereas BGP-486—of the same species—increased root dry weight by up to 43% and root tissue density by up to 30% in all inoculated treatments. In *Paspalum regnellii* BGP-112, seed inoculation produced stronger root responses than foliar spray, increasing root dry weight by up to 38% and root area by up to 40%. In *Paspalum rojasii* BGP-149, inoculation induced root architectural remodeling without increasing root biomass—root area increased by up to 92% and total root length by up to 97%, relative to the control—indicating an efficient root-foraging strategy. *P. fluorescens* favored root-foraging traits and tissue density, whereas *A. brasilense* preferentially stimulated root-hair elongation. These accession-level contrasts, rather than a single generalizable trend, are the main contribution of this work: they support tailored, genotype-specific PGPB strategies for sustainable tropical pastures.

## 1. Introduction

Brazil is one of the world’s leading beef producers and exporters [1,2]; with 195.5 million head of cattle, Brazil has consolidated its position as the country with the largest commercial cattle herd in the world [2]. Cattle production is predominantly pasture-based, occupying approximately 166 million hectares (Mha), an area more than twice the size of that used for agriculture in the country [3,4,5]. Despite its economic importance, the sector faces a persistent challenge: pasture degradation. Recent estimates indicate that approximately 58.8% of Brazilian pastures show some degree of degradation [6], a process characterized by reduced cattle-carrying capacity, soil compaction, and loss of forage vigor [7,8,9]. Degraded pastures also increase pressure on native vegetation, as new areas are opened to compensate for productivity losses [10].

Nitrogen (N) deficiency is the main factor driving pasture degradation in tropical soils, as N is usually the primary nutrient limiting forage biomass production [11]. The biological basis for an alternative N supply to tropical grasses was established by Dr. Johanna Döbereiner in the 1970s, when associative interactions between diazotrophic bacteria and roots of tropical forage grasses were described [12,13]. Despite this knowledge, the conventional approach still relies heavily on synthetic N fertilizers, which generate high input costs and environmental risks, including emissions of nitrous oxide (N_2_O), a greenhouse gas with a global warming potential approximately 273 times that of CO_2_ over a 100-year horizon [14], and nitrate contamination of water bodies [15].

The use of inoculants formulated with plant growth-promoting bacteria (PGPB) represents a biotechnological strategy derived from the knowledge accumulated since Döbereiner’s first report. PGPB may promote plant growth through several microbial processes, including biological nitrogen fixation (BNF), synthesis of phytohormones such as indole-3-acetic acid (IAA), ACC-deaminase (1-aminocyclopropane-1-carboxylate deaminase) activity, phosphate solubilization, siderophore production, and induction of resistance to biotic and abiotic stresses [4,16,17,18]. These mechanisms may improve N and P nutrition directly and indirectly by modifying root architecture, increasing the plant’s capacity to acquire water and nutrients [19,20].

Among the PGPB, *Azospirillum brasilense* is the most studied species for grass crops in Brazil, with an emphasis on strains Ab-V5 (=CNPSo 2083) and Ab-V6 (=CNPSo 2084) [15]. Besides biological nitrogen fixation (BNF), the reported effects of *A. brasilense* are mainly mediated by IAA synthesis, which promotes lateral root proliferation, increases root hair density and length, and favors greater root volume and surface area, improving water and nutrient uptake [16,19,20,21]. A meta-analysis of 103 field trials with maize (*Zea mays* L.) inoculated with strains Ab-V5 and Ab-V6 reported average increases of 12.1% in root mass and 5.4% in grain yield across diverse Brazilian conditions [22]. In forage grasses of the genus *Urochloa*, which occupy approximately 86 Mha of Brazilian pastures, inoculation with Ab-V5 and Ab-V6 via seeds or leaf spray increased forage biomass by an average of 16.8% and N content by up to 20.7% [8,20].

The complementary use of *Pseudomonas fluorescens* expands the PGPB toolkit for grass inoculation. Unlike *A. brasilense* strains Ab-V5 and Ab-V6, *P. fluorescens* strain CNPSo 2719 possesses ACC-deaminase activity, reducing the ethylene-mediated inhibition of root elongation, as well as siderophore production and inorganic phosphate solubilization, properties confirmed by Hungria et al. [20] and, more recently, by Vasques et al. [18], who screened these strains for agronomically relevant traits. Phosphate solubilization is particularly relevant in tropical soils with high phosphorus (P) fixation capacity [23]. Other PGPB, such as *Bacillus*, can contribute to these mechanisms, as well as others, such as biocontrol of pests and diseases [4,18,24]. For root systems established in nutrient-poor tropical soils with high P-fixation, such as those where *Paspalum* naturally occurs, ACC-deaminase activity and phosphate solubilization are expected to be particularly advantageous, as they can directly relieve two major constraints on early root growth: ethylene-mediated inhibition of elongation and P scarcity.

*Paspalum* represents a major genus within South American tropical and subtropical grasslands, comprising about 350 species [25], of which approximately 220 are native to Brazil [26]. The genus is adapted to diverse edaphoclimatic conditions and ecosystems [27]. Species of *Paspalum* are well represented in rangelands used for cattle production, as well as cultivated for forage and turf [28].

PGPB–plant interactions are host-specific; in general, results obtained for one forage genus cannot be directly extrapolated to others [16,29,30], and *Paspalum* has been little studied regarding its response to inoculation with PGPB, in contrast with the literature available for other tropical forage genera. Despite decades of research on PGPB–grass interactions in Brazil, virtually no studies in recent decades have addressed *Paspalum*, and none have evaluated the germplasm accessions used in this study. This emphasizes the gap that mirrors the broader scarcity of inoculation studies in grass pastures worldwide, where prior work by our group has already shown that responses differ markedly in genera and species of *Urochloa* and *Panicum* [5,8,20,30].

One of the few studies available identified that rhizobacterial communities associated with *Paspalum* were dominated by *Bacillus*, *Rhizobium* and *Paraburkholderia*, differing from those of *Urochloa* [31]. In addition, the responses to inoculation were highly genotype-dependent: inoculation with *Azospirillum baldaniorum* Sp245 increased shoot dry matter by up to 53% in *Paspalum regnellii* but had negligible effects in *Paspalum malacophyllum* and *Paspalum atratum*, reinforcing that cross-genus extrapolation is inadequate, highlighting the need for studies within the genus. Although PGPB effects on *Urochloa*—the most extensively studied forage genus in Brazil—are better characterized, including consistent gains in forage biomass and root growth following seed or foliar inoculation with *A. brasilense* [8,20], no equivalent framework exists for *Paspalum*, and it remains unclear whether the mechanisms and inoculation strategies effective in *Urochloa* can be transferred to this genus. This knowledge gap is the central rationale for the present study, which represents, to our knowledge, the first systematic evaluation of PGPB effects on root architecture across multiple *Paspalum* accessions.

To increase the still-limited knowledge about the PGPB–*Paspalum* interaction, we evaluated, under controlled axenic greenhouse conditions, the root morphology and shoot growth responses of four *Paspalum* accessions to inoculation with strains Ab-V5 and Ab-V6 of *A. brasilense* and CNPSo 2719 of *P. fluorescens*, applied via seed inoculation or leaf spray. We hypothesized that root and shoot growth responses of *Paspalum* to PGPB inoculation are genotype-dependent, varying according to the accession, the inoculation method (seed vs. leaf spray), and the bacterial strain used.

## 2. Results

### 2.1. Root System Responses

**Figure 1:**
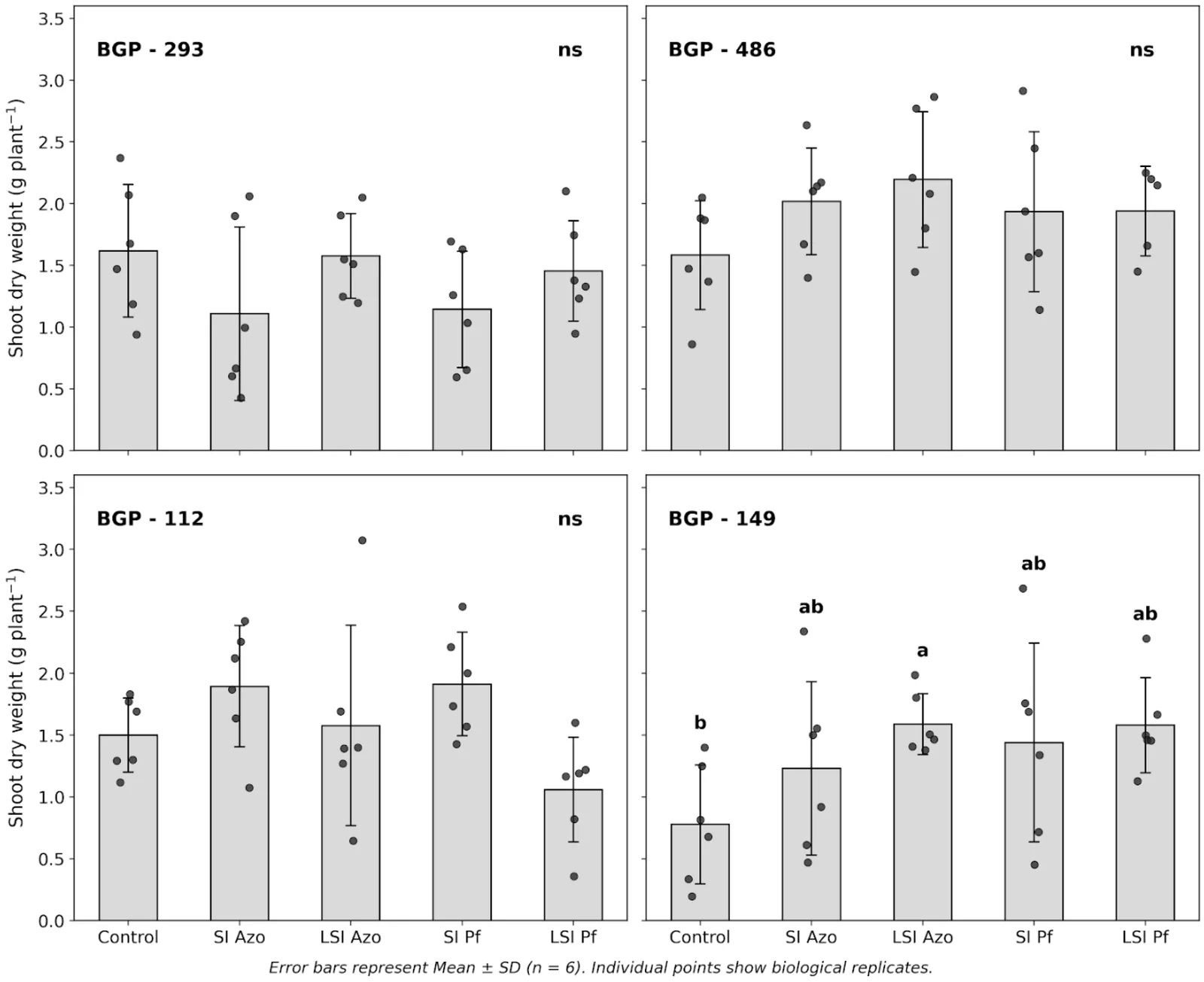
Shoot dry weight of four *Paspalum* spp. accessions (*Paspalum malacophyllum* BGP-293 and BGP-486, *Paspalum regnellii* BGP-112, and *Paspalum rojasii* BGP-149) submitted to different inoculation treatments with *Azospirillum brasilense* (CNPSo 2083 + CNPSo 2084) or *Pseudomonas fluorescens* (CNPSo 2719) via seeds or leaf spray, in a greenhouse assay under axenic conditions (35 DAE). Vertical bars = standard deviation (n = 6). Means followed by distinct letters differ by Duncan’s test at *p* < 0.05; ns = not significant. Control = non-inoculated; SI Azo = seed inoculation with *A. brasilense*; LSI Azo = leaf-spray inoculation with *A. brasilense*; SI Pf = seed inoculation with *P. fluorescens*; LSI Pf = leaf-spray inoculation with *P. fluorescens*.

The root morphological parameters evaluated at 35 days after emergence (DAE) revealed that the response of *Paspalum* accessions to inoculation with *A. brasilense* strains CNPSo 2083 and CNPSo 2084 and *P. fluorescens* strain CNPSo 2719 varied according to host genotype and inoculation method (Table 1; Figure 1).

Root dry weight (RDW) of *P. malacophyllum* BGP-293 varied from 0.46 to 0.67 g plant^−1^ with no statistical difference between treatments, a result that extended to volume, total length, surface area, tissue density, and all parameters related to root hairs and branching. In contrast, accession BGP-486, also belonging to *P. malacophyllum*, responded positively to PGPB inoculation. All treatments significantly increased RDW compared to the non-inoculated control (0.93 vs. 1.26–1.33 g plant^−1^), while simultaneously increasing root tissue density (RTD) from 0.10 to 0.12–0.13 g cm^−3^ and reducing specific root length (SRL) in the LSI Pf treatment (102 vs. 126–135 cm g^−1^). Volume, total length, root area, and root hair parameters did not differ significantly between treatments (Table 1).

*Paspalum regnellii* BGP-112 showed significant effects on RDW, root volume (RV), total root length (TRL), root area (RA), incidence and length of root hairs, and number of branches but only for specific treatment × variable combinations (Table 1). Seed inoculation with *A. brasilense* (SI Azo) and with *P. fluorescens* (SI Pf) produced consistently superior responses to the respective foliar spray treatments: SI Azo and SI Pf achieved higher RDW, RV, TRL, and RA (Table 1). The number of branches was also significantly higher in SI Azo and SI Pf compared to the control and LSI Pf, with LSI Azo in an intermediate position. Taken altogether, seed inoculation generally produced stronger root responses than foliar spray, particularly in BGP-112, where the seed-applied treatments (SI Azo, SI Pf) outperformed their foliar-spray counterparts (LSI Azo, LSI Pf) across most root traits.

Regarding root hairs in BGP-112, incidence was significantly higher in all four inoculation treatments compared to the control. LSI Pf (53%) showed the highest incidence, followed by SI Pf (46%) and SI Azo/LSI Azo (37–39%) vs. the control (25%). Root hair length was significantly greater in SI Azo (210 µm) and LSI Azo (203 µm) than in the control (172 µm).

The genotype *P. rojasii* BGP-149 presented architectural remodeling of the root system without a corresponding increase in root dry weight (Table 1). All four inoculation treatments significantly increased RV (6.57–7.38 cm^3^ plant^−1^ vs. 3.89 cm^3^ in the control), TRL (115–138 vs. 70.1 m plant^−1^), and RA (901–1124 cm^2^ plant^−1^ vs. 585 cm^2^ plant^−1^). Specific root length (SRL) was significantly higher than the control (132 cm g^−1^) for SI Azo (184), LSI Azo (175), and LSI Pf (192). The number of branches was significantly higher in LSI Azo (35,589), SI Pf (37,338), and LSI Pf (32,539) compared to the control (18,828).

Across all four accessions, the results converge on a single central finding: PGPB effects on root architecture in *Paspalum* are strongly accession-specific rather than uniform, ranging from negligible (BGP-293) to biomass gains (BGP-486), superior root development following seed relative to foliar inoculation (BGP-112), and architectural remodeling without biomass gain (BGP-149).

### 2.2. Shoot Biomass

Regarding shoot evaluation, dry mass (SDW) did not differ between inoculation treatments for accessions BGP-293 (*P. malacophyllum*), BGP-486 (*P. malacophyllum*), and BGP-112 (*P. regnellii*) (Figure 1), indicating that the effects of PGPB on these genotypes were predominantly restricted to the root system. For BGP-149 (*P. rojasii*), foliar spray with *A. brasilense* (LSI Azo) was the only treatment that significantly increased SDW compared to the non-inoculated control; the other inoculated treatments (SI Azo, SI Pf, and LSI Pf) presented intermediate values, with no statistical difference compared to the control or LSI Azo (Figure 1).

## 3. Discussion

The results obtained in this study confirm that PGPB responses in *Paspalum* are highly host-specific and dependent on both the bacterial strain and the inoculation method. This host specificity is presumably consistent with the importance of root exudate composition, chemotactic signals, and receptor-mediated recognition in PGPR–plant interactions [16,31], although these mechanisms were not directly measured in the present study.

The absence of response in *P. malacophyllum* BGP-293 is in agreement with Amaral et al. [31], who reported small or absent effects of PGPR inoculation on the same accession. The lack of response may reflect incompatibility between the chemistry of the root exudates of this accession and the chemotactic requirements of strains CNPSo 2083, CNPSo 2084, and CNPSo 2719 for effective rhizosphere colonization and phytohormone delivery [15,32], although this was not measured directly and remains a plausible explanation rather than a demonstrated mechanism.

The contrasting responses between BGP-293 (no response) and BGP-486 (positive response), both belonging to *P. malacophyllum*, evidence that responsiveness to PGPR operates at the accession level and not at the species level, likely reflecting differences in root exudate composition [32,33]. The increase in RTD in all inoculated treatments with BGP-486 is agronomically relevant for tropical pasture management, as this parameter is generally associated with the degree of cell wall thickening and lignification of root tissues, attributes associated with mechanical resistance to soil compaction and greater root longevity under water stress [34,35].

The superiority of seed inoculation over foliar spray for the root attributes of BGP-112 may be attributed to early colonization of the seed surface during germination [36]. Bacteria applied directly to the seed could interact with exudates induced by high-density germination, potentially enabling the delivery of IAA to the root meristem at the initial stages of development [37]. IAA, produced by more than 80% of rhizospheric bacteria, including the genus *Pseudomonas*, acts as an inducer of cell division and a stimulant for the formation of lateral and adventitious roots [38], which may explain why the SI Pf treatment reached the highest value for number of branches (44,893 per plant).

The root hair responses also suggest different mechanisms among bacterial treatments. The high incidence observed in the LSI Pf treatment suggests a possible reallocation of resources from structural root biomass to the soil exploration surface, potentially mediated in part by the ACC-deaminase activity of CNPSo 2719, which is known to reduce ethylene levels that, at high concentrations, would inhibit hair initiation [39]. ACC-deaminase and IAA synthesis by the strains used in our study were confirmed in vitro by Vasques et al. [18]. In contrast, the increase in root hair length in SI Azo and LSI Azo suggests that *A. brasilense* may have promoted hair elongation through IAA-dependent pathways. Zhao et al. [40] reported a 1.5- and 4.27-fold increase in root hair density and length in *Arabidopsis* roots inoculated with *A. brasilense*, corroborating the findings of the present study. Root hairs constitute the main sites for the absorption of low-mobility nutrients, such as P, and their proliferation expands the effective absorption surface [34].

The dissociation between stable root biomass and architectural expansion in BGP-149 characterizes a root foraging strategy, through which plants increase the efficiency of soil exploration per unit of biomass invested [41]. By producing finer (higher SRL) and more branched roots without an increase in total root mass, BGP-149 with PGPR inoculation increases soil coverage per unit of root biomass. This phenotype is expected to be especially advantageous for P acquisition, a nutrient accessible only in the immediate vicinity of root surfaces in tropical soils with high P fixation [41], although P acquisition was not directly measured in this study. The expansion of the root area from 585 to more than 1000 cm^2^ plant^−1^ represents an increase of approximately 70%.

The numerically greater architectural response in LSI Pf relative to LSI Azo—although the two treatments did not differ significantly for BGP-149—suggests that the ACC-deaminase and phosphate solubilization activities of *P. fluorescens* CNPSo 2719 [20] may contribute to mechanisms beyond those of *A. brasilense*. By potentially reducing the accumulation of stress ethylene, ACC-deaminase could prevent the root thickening response that this hormone would otherwise induce, maintaining a foraging architecture with fine roots and high SRL [17]. Mobilized phosphate could also reinforce the foraging phenotype by providing a nutritional signal that sustains lateral branching [42].

The absence of shoot response in accessions with a significant root response, such as BGP-112, suggests that, at least during the short evaluation period (35 DAE), the benefits of inoculation manifest first in root system architecture, with effects on shoot biomass possibly becoming more pronounced at later phenological stages or under nutritional stress conditions. For BGP-149, the selective increase in SDW after foliar spray with *A. brasilense* may be related to the ability of Ab-V5 and Ab-V6 to increase phytohormone concentrations in shoots, as demonstrated in maize [42].

Because assays were conducted under axenic conditions, in a substrate free of microbial competition, and over a relatively short period (35 DAE), the present findings should be interpreted primarily as a controlled physiological screening rather than as a direct prediction of field performance. Axenic greenhouse conditions remove the confounding effects of soil microbial competition, nutrient heterogeneity, and abiotic stress, allowing the direct physiological effects of the inoculated strains to be isolated, but they do not reproduce the complexity of established pastures, including soil compaction, competition from native rhizosphere communities, and heterogeneous nutrient distribution. Under the 500 cm^3^ jar volume and 50% N Hoagland’s solution used here, it is also possible that N and P availability became more limited over time for some accessions, which could partly explain the more restricted shoot responses observed in three out of the four genotypes. Rather than a limitation that weakens the conclusions, this first stage is deliberately designed to identify, among the accession × strain × application-method combinations tested, the most promising candidates to advance to field validation trials, where soil type, grazing pressure, and long-term rhizosphere persistence of the inoculated strains can be properly assessed. Additionally, a single non-inoculated control was used for all treatments, and re-isolation of the inoculated strains from roots and shoots was also not performed. A further methodological consideration concerns total root length (TRL) measurement: because the line-intersection method was applied manually on grid plates, and some root systems reached lengths above 100 m per plant (e.g., BGP-149, up to 138 m plant^−1^), care was taken to standardize root dispersion and intersection counting across samples to minimize operator-related variability; nonetheless, some degree of measurement variability inherent to manual line-intersection counting cannot be excluded, particularly for the most extensive root systems.

This study should be regarded as the first stage of a broader research effort. However, this first step is very important to choose treatments and parameters to be considered in the following steps. Field experiments are expensive and, in the case of grass forages, involve intensive and long-term conduct. Therefore, a preliminary screen is very useful. In addition, root parameters are probably the most important feature for successful pasture establishment and, under field conditions, evaluation of most root parameters shown in this study is less precise than under controlled conditions.

## 4. Materials and Methods

### 4.1. Inoculant Preparation

All strains are deposited at the “Diazotrophic and Plant Growth-Promoting Bacteria Culture Collection of Embrapa Soja” (WFCC #1213, WDCM #1054) in Londrina, Paraná State, Brazil. Inoculants with the strains CNPSo 2083 (=Ab-V5) and CNPSo 2084 (=Ab-V6) of *A. brasilense* were prepared independently in DYGS broth [43]. After growing at 180 rpm, 28 °C, for 120 h, corresponding to the stationary phase, strains reached the concentration of 10^9^ colony-forming units (CFUs) mL^−1^. Cultures were then mixed, and the concentration was adjusted to 2.0 × 10^5^ CFUs mL^−1^. Strains CNPSo 2083 and CNPSo 2084 were mixed because the great majority of commercial inoculants with *A. brasilense* in Brazil carry both strains [15]. Under field conditions, a proper concentration of the strains would be of 10^8^ and 10^9^ CFUs mL^−1^, given the high rate of cell death typically observed after application; however, under the optimal controlled conditions of a greenhouse assay, a lower concentration correlates better with the effective viable population reaching the rhizosphere under field conditions. Inoculant with the strain CNPSo 2719 (=CCTB 03) of *P. fluorescens* was prepared in TSB broth (Tryptic Soy Broth, Acumedia, Lansing, MI, USA, 30 g L^−1^) and, after growing for 120 h at 180 rpm and 28 °C, reached the stationary phase of 10^9^ CFUs mL^−1^. Concentration of viable cells was also adjusted to 2.0 × 10^5^ CFUs mL^−1^. The adjustments to inoculant concentrations were made based on bacterial growth curves with the correspondence of CFUs with optical density (O.D.) currently used in the laboratory for these strains and then confirmed by CFU counting. Strain viability was further confirmed by cell counting using the plate spread method in both DYGS and TSB media, and IAA production and ACC-deaminase activity of these same strains were previously confirmed in vitro by Vasques et al. [18].

### 4.2. Experimental Design and Plant Growth Conditions

A greenhouse experiment was carried out under axenic conditions at Embrapa Soja, Londrina, Paraná State, southern Brazil (23°11′ S, 51°11′ W), with average temperatures of 28/20 °C (day/night), average relative humidity of 78/60% (day/night), and a photoperiod of 12/12 h. Four accessions of *Paspalum* spp. were evaluated: *Paspalum malacophyllum* BGP-293 and BGP-486, *Paspalum regnellii* BGP-112, and *Paspalum rojasii* BGP-149. From here, the accessions will be referred to as BGP-293, BGP-486, BGP-112, and BGP-149. The seeds were provided by the germplasm bank of Embrapa Pecuária Sudeste, São Carlos, São Paulo State, Brazil. Plants were cultivated in modified Leonard jars [44] with 500 cm^3^ volume, filled with a sterile substrate composed of a 3:1 mix (*v*/*v*) of coarse sand and milled coal, respectively, the latter consisting of regular eucalyptus charcoal, ground to an average particle size of 2 mm, and receiving sterile Hoagland’s nutrient solution and pH 6.5–7.0 [45], except for a reduction of 50% of the N concentration. In Leonard jars [44], nutrient solution was always available to the plants by filling the lower recipient.

For all accessions, the treatments consisted of (i) a non-inoculated control (Control); (ii) seed inoculation with *A. brasilense* strains CNPSo 2083 and CNPSo 2084 (SI Azo); (iii) leaf-spray inoculation with CNPSo 2083 and CNPSo 2084 (LSI Azo); (iv) seed inoculation with *P. fluorescens* strain CNPSo 2719 (SI Pf); and (v) leaf-spray inoculation with CNPSo 2719 (LSI Pf). The experiment was performed in a completely randomized design with six replicates per treatment.

Sowing was performed in early November 2021. Before sowing, seeds were surface-disinfested by immersion in 70% ethanol for 1 min, followed by 4% sodium hypochlorite for 3 min and six consecutive rinses with sterilized water, following the protocol described by Hungria et al. [46]. Six seeds were sown per jar, and five days after emergence (DAE) seedlings were thinned to one plant per jar. For seed inoculation, 1 mL of each inoculant was added at the sowing hole, providing 2.0 × 10^5^ CFUs sowing hole^−1^. Inoculation via leaf spray was performed 13 DAE using an aerograph atomizer previously calibrated to deliver 1 mL of inoculant per plant (2.0 × 10^5^ CFUs plant^−1^) [20], according to the treatment. The equipment used for foliar application was sterilized before each application, with 70% ethanol, followed by 4% sodium hypochlorite solution and two rinses with sterile water, to prevent cross-contamination between treatments. The surfaces of the jars were covered with aluminum foil at the time of leaf spraying to prevent the inoculants from reaching the substrate.

### 4.3. Shoot Biomass and Root Trait Assessments

Thirty-five DAE, plants were removed from the jars, and shoots and roots were separated. Shoots were oven-dried at 60 °C until constant weight to assess shoot dry weight (SDW). Root systems were washed in tap water, and root volumes (RVs) were obtained by immersing and displacing water in graduated cylinders. Total root length (TRL) was assessed in fresh root systems by the modified line-intersection method, using plates with 1 × 1 cm grid squares where fresh roots were randomly arranged and their intercepts with the grid lines were counted; TRL was estimated by the formula TRL = N × 0.7857, where N represents the number of intercepts, and 0.7857 is the length conversion factor [47]. Root mean diameter (RMD) was determined by the formula 2 × [RV/(TRL × π)]^0.5^ [19]. Root system area (RA) was calculated by the formula π × RMD × TRL [30].

Subsamples with approximately 0.30 g of fresh fine roots and <2.0 mm diameter were taken from each root system, stored in FAA solution (5% formaldehyde, 5% acetic acid, 90% ethanol-70%, v:v:v), and used for the assessment of number of root branches and root-hair incidence. After the analyses, the roots were dried and included in consideration of the final RDW measurements. The number of root branches (RBs) was assessed under a stereomicroscope at 20× magnification by counting all lateral roots in the fine-root subsamples and was estimated as follows: RB = (number of lateral roots in the fine-root subsamples × root fresh weight)/0.30. Root-hair length (RHL) was assessed by measuring 100 root hairs in at least 30 fine root segments per sample using a microscope at 100× magnification with an ocular micrometer [19]. The roots used to assess root hairs were fine roots (less than 2.0 mm in diameter). The root system was vertically divided into three sections, and fine roots were collected from its middle and basal thirds, with the basal third located near the root–shoot transition zone. Root-hair incidence (RHI) was determined by the presence or absence of root hairs on 100 fine-root intersections per sample using a microscope at 100× magnification and the gridline method [48].

Roots were oven-dried at 60 °C until constant dry weight to assess root dry weight (RDW). Specific root length (SRL) was determined by the ratio TRL/RDW, and root-tissue density (RTD) was calculated by the ratio RDW/RV [19].

### 4.4. Statistical Analysis

For the root and shoot parameters, data were first tested for normality by Shapiro–Wilk’s test and homoscedasticity by O’Neill and Mathews’ test [49]. Root-hair incidence, expressed as percentage, was transformed to arcsine (×/100)^0.5^ before analysis. Means were analyzed by one-way ANOVA, applied separately within each genotype, followed by Duncan’s test. This approach—analyzing treatment effects within each accession separately, rather than pooling accessions in a single factorial model—was chosen because the primary objective of this study was to characterize genotype-specific responses at the accession level; a pooled analysis with a genotype × treatment interaction term would obscure within-genotype contrasts that are of direct agronomic relevance for genotype-specific inoculant recommendations. The same analytical approach has been used in previous studies from our group on PGPB–grass interactions, including a study published in this journal [30], as it provides a clearer view of genotype-specific responses. Duncan’s test was adopted in accordance with Normative Instruction No. 13 of the Brazilian Ministry of Agriculture, Livestock and Food Supply (24 March 2011) [50], which specifies statistical procedures for inoculant registration and evaluation in Brazil. All tests were performed in Rbio v.171/2022 software [51], considering α = 5%.

## 5. Conclusions

This study demonstrates that Paspalum responses to inoculation with PGPB are highly accession-specific rather than following a single overarching pattern, indicating that species-level generalizations are inadequate for technical recommendations. The distinct responsiveness between accessions of *P. malacophyllum* (BGP-293 and BGP-486) highlights the necessity for targeted genotype screening before inoculant adoption. Furthermore, seed inoculation was generally more effective than leaf-spray applications in modifying root architecture, particularly in BGP-112, although this trend was not uniform across all accessions and root indicators and did not extend, for instance, to the biomass response of BGP-149, as early bacterial colonization is crucial to maximize morphological benefits. The evaluated bacterial strains also demonstrated distinct and complementary effects on root remodeling: *P. fluorescens* CNPSo 2719 increased root tissue density and drove strong foraging traits, whereas *A. brasilense* strains primarily promoted root-hair elongation. The pronounced root foraging phenotype observed in *P. rojasii* (BGP-149), characterized by expanded root volume, specific root length, and surface area without an increase in structural biomass, represents a highly advantageous trait for improving nutrient acquisition in low-P tropical soils and degraded pastures.

Early-stage root system responses, as characterized here, are directly relevant to the establishment and persistence of tropical pastures, particularly under water-limiting conditions, since root architecture developed at the seedling stage can influence a plant’s capacity to explore soil resources throughout later phenological stages. These findings therefore provide a foundation for breeding programs and for the future development of genotype-specific microbial inoculants aimed at improving the establishment and long-term resilience of tropical pastures.

Together, these findings highlight that matching specific *Paspalum* accessions with appropriate bacterial strains and inoculation methods is a viable biotechnological strategy to improve pasture sustainability. As the present results were obtained under controlled axenic conditions over a relatively short evaluation period, they should be regarded as an initial screening stage, warranting further validation under field conditions—including different soil types, grazing intensities, and assessment of the long-term persistence of rhizosphere colonization by the inoculated strains—to confirm whether the observed root architectural changes translate into improved forage productivity and pasture longevity.

## Figures and Tables

**Table 1:** Root traits of four *Paspalum* spp. accessions grown under greenhouse conditions and receiving different inoculation treatments with *Azospirillum brasilense* (CNPSo 2083 + CNPSo 2084) or *Pseudomonas fluorescens* (CNPSo 2719), via seed inoculation or leaf spray. Roots were sampled 35 days after plant emergence.

Treatments	Dry Weight	Volume	Total Length	Specific Length	Area	Mean Diameter	Tissue Density	Hair Incidence	Hair Length	Number of Branches
	(g plant^−1^)	(cm^3^ plant^−1^)	(m plant^−1^)	(cm g^−1^)	(cm^2^ plant^−1^)	(µm)	(g cm^−3^)	(%)	(µm)	(per plant)
*Paspalum malacophyllum BGP-293*										
Control	0.48 ns	5.11 ns	76.1 ns	159 ns	697 ns	292 ns	0.09 ns	47 ns	172 ns	17,940 ns
SI Azo	0.49	4.95	63.6	131	513	290	0.10	57	170	16,004
LSI Azo	0.67	6.06	92.7	137	804	276	0.11	56	171	23,144
SI Pf	0.46	5.00	66.0	145	544	271	0.09	53	173	15,269
LSI Pf	0.53	4.60	77.4	152	705	269	0.12	54	178	20,420
*p* value	*0.170*	*0.425*	*0.201*	*0.789*	*0.327*	*0.517*	*0.146*	*0.832*	*0.872*	*0.294*
CV (%)	28.1	27.1	29.7	25.9	22.6	10.3	17.6	20.7	8.38	37.4
										
*Paspalum malacophyllum BGP-486*										
Control	0.93 b	8.75 ns	124 ns	132 a	1203 ns	290 ns	0.10 b	46 ns	189 ns	35,138 ns
SI Azo	1.31 a	10.7	171	135 a	1516	284	0.12 a	47	178	42,390
LSI Azo	1.26 a	10.5	157	126 a	1439	294	0.12 a	39	175	40,155
SI Pf	1.27 a	10.7	164	129 a	1483	288	0.12 a	49	189	41,935
LSI Pf	1.33 a	10.3	134	102 b	1299	313	0.13 a	39	186	37,212
*p* value	*0.048*	*0.515*	*0.203*	*0.031*	*0.280*	*0.200*	*0.035*	*0.388*	*0.358*	*0.660*
CV (%)	26.2	20.8	23.1	15.8	20.9	7.41	10.9	16.9	7.98	24.9
										
*Paspalum regnellii BGP-112*										
Control	0.98 b	7.49 ab	108 bc	110 ns	1007 b	300 ns	0.13 ns	25 c	172 b	29,180 bc
SI Azo	1.35 a	9.91 a	160 a	122	1401 a	279	0.14	37 b	210 a	41,665 a
LSI Azo	0.96 b	7.40 ab	130 ab	137	1095 ab	271	0.14	39 b	203 a	36,726 ab
SI Pf	1.27 a	9.88 a	159 a	124	1405 a	282	0.13	46 ab	183 ab	44,893 a
LSI Pf	0.83 b	5.46 b	95.6 c	118	743 b	295	0.11	53 a	194 ab	26,883 c
*p* value	*<0.001*	*0.008*	*0.003*	*0.464*	*0.002*	*0.249*	*0.158*	*<0.001*	*0.043*	*0.048*
CV (%)	21.0	27.5	28.3	25.1	26.3	8.46	26.8	24.8	7.96	19.3
										
*Paspalum rojasii BGP-149*										
Control	0.53 ns	3.89 b	70.1 b	132 b	585 b	272 ns	0.09 ns	30 ns	151 ns	18,828 b
SI Azo	0.64	6.57 a	120 a	184 a	957 a	253	0.10	30	160	27,868 ab
LSI Azo	0.71	6.89 a	128 a	175 a	1060 a	266	0.11	39	153	35,589 a
SI Pf	0.74	7.19 a	115 a	160 ab	901 a	245	0.10	32	150	37,338 a
LSI Pf	0.72	7.38 a	138 a	192 a	1124 a	261	0.09	34	155	32,539 a
*p* value	*0.438*	*0.001*	*0.042*	*0.040*	*0.034*	*0.091*	*0.770*	*0.844*	*0.788*	*0.032*
CV (%)	31.0	20.6	32.8	19.9	21.8	10.8	19.2	28.4	11.4	33.7

^a^ Control = non-inoculated; SI Azo = seed inoculation with *Azospirillum brasilense* strains CNPSo 2083 and CNPSo 2084; LSI Azo = leaf-spray inoculation with CNPSo 2083 and CNPSo 2084; SI Pf = seed inoculation with *Pseudomonas fluorescens* strain CNPSo 2719; LSI Pf = leaf-spray inoculation with CNPSo 2719. ^b^ Data represent the means of six replicates. Means followed by distinct letters differ from each other by Duncan’s test at *p* < 0.05; ns = not significant.

## Data Availability

The raw data supporting the conclusions of this article will be made available by the authors on request.

## Abbreviations

The following abbreviations are used in this manuscript: ACC, 1-aminocyclopropane-1-carboxylate; BNF, biological nitrogen fixation; CFUs, colony-forming units; CV, coefficient of variation; DAE, days after emergence; IAA, indole-3-acetic acid; LSI, leaf-spray inoculation; PGPB, plant growth-promoting bacteria; PGPR, plant growth-promoting rhizobacteria; RA, root area; RBs, root branches; RDW, root dry weight; RHI, root-hair incidence; RHL, root-hair length; RMD, root mean diameter; RTD, root tissue density; RV, root volume; SDW, shoot dry weight; SI, seed inoculation; SRL, specific root length; TRL, total root length.
